# Informing National Health Service patients about participation in clinical research: A comparison of opt-in and opt-out approaches across the United Kingdom

**DOI:** 10.1177/0004867420973261

**Published:** 2020-11-21

**Authors:** Catherine Henshall, Jennifer Potts, Sophie Walker, Mark Hancock, Mark Underwood, Nick Broughton, Roger Ede, Catherine Kernot, Lorcan O’Neill, John R Geddes, Andrea Cipriani

**Affiliations:** 1 Faculty of Health and Life Sciences, Oxford Brookes University, Oxford, UK; 2 Oxford Health NHS Foundation Trust, Warneford Hospital, Oxford, UK; 3 Department of Psychiatry, University of Oxford, Oxford, UK

**Keywords:** Barriers to recruitment, mental health research opportunities, equity, opt-in, opt-out

## Abstract

**Objective:**

Recruitment to clinical research in the National Health Service remains challenging. One barrier is accessing patients to discuss research participation. Two general approaches are used in the United Kingdom to facilitate this: an ‘opt-in’ approach (when clinicians communicate research opportunities to patients) and an ‘opt-out’ approach (all patients have the right to be informed of relevant research opportunities). No evidence-based data are available, however, to inform the decision about which approach is preferable. This study aimed to collect information from ‘opt-in’ and ‘opt-out’ Trusts and identify which of the two approaches is optimal for ensuring National Health Service patients are given opportunities to discuss research participation.

**Method:**

This sequential mixed methods study comprised three phases: (1) an Appreciative Inquiry across UK Trusts, (2) online surveys and (3) focus groups with National Health Service staff and patients at a representative mental health Trust.

**Results:**

The study was conducted between June and October 2019. Out of seven National Health Service Mental Health Trusts contacted (three ‘opt-out’ and four ‘opt-in’), only four took part in phase 1 of the study and three of them were ‘opt-out’ Trusts. Benefits of an ‘opt-out’ approach included greater inclusivity of patients and the removal of research gatekeepers, while the involvement of research-active clinicians and established patient–clinician relationships were cited as important to ‘opt-in’ success. Phases 2 and 3 were conducted at a different Trust (Oxford Health NHS Foundation Trust) which was using an ‘opt-in’ approach. Of 333 staff and member survey responders, 267 (80.2%) favoured moving to an ‘opt-out’ approach (phase 2). Nineteen staff and 16 patients and carers participated in focus groups (phase 3). Concern was raised by staff regarding the lack of time for clinical research, with clinical work taking precedence over research; patients were concerned about a lack of research activity; all considered research to be beneficial and were supportive of a move to ‘opt-out’.

**Conclusion:**

Findings suggest that ‘opt-out’ is more beneficial than ‘opt-in’, with the potential to vastly increase patient access to research opportunities and to enable greater equality of information provision for currently marginalised groups. This should ensure that healthcare research is more representative of the entire population, including those with a mental health diagnosis.

## Introduction

Globally, patients who participate in research have improved clinical outcomes (Ozdemir et al., 2015) and greater satisfaction with care (Jonker et al., 2020). Moreover, research-active clinical services within healthcare organisations have lower mortality rates and produce higher quality care outcomes (3), highlighting the positive effects of healthcare research at an international level (Royal College of Physicians, 2020).

In the United Kingdom, two main approaches are taken to inform patients about research within the National Health Service (NHS). First, ‘opt-in’ approach relies on clinicians communicating research opportunities to patients, and obtaining their permission to contact them about participation. Second, ‘opt-out’ approach is underpinned by the philosophy that all patients have the right to be informed of relevant research opportunities to enable them to make an informed decision about participation.

Different NHS Trusts employ different approaches, with many adopting the ‘opt-in’ approach. However, recruiting participants to clinical research holds many challenges for staff within the NHS (Jones and Cipriani, 2019), including a lack of time and decreased research awareness and engagement. In addition, researchers are often reliant on clinicians, as ‘gatekeepers’, to recruit participants to studies (Borschmann et al., 2014), which can limit uptake. This is especially true in mental health, as illustrated by a recent pilot study of an ‘opt-in’ approach at Oxford Health NHS Foundation Trust (OHFT) which found that only 11% (*n* = 197) of patients had participated in a research discussion with their clinician (Walker et al., 2020). This demonstrates that the current way of recruiting participants to clinical research is not effective or equitable in ensuring patients are made aware of opportunities to have better healthcare.

In order to address this equity issue, some Trusts have moved from an ‘opt-in’ to an ‘opt-out’ approach (National Institute for Health Research, 2020); however, no evidence-based data are available to inform the decision about which approach is preferable and what sort of framework can be used to implement these approaches. These are important questions, the answers to which are likely to determine future research, and for this reason, we designed a study aimed to identify whether the ‘opt-in’ or ‘opt-out’ approach was optimal for ensuring all patients are made aware of mental health research opportunities. The study was deliberately sequential in its design to allow national forerunners guidance to inform local practice.

## Methods

This sequential mixed methods study comprised three phases: (1) an Appreciative Inquiry across some UK Trusts, (2) an online survey with NHS staff and members and (3) focus groups with NHS patients and staff at a representative mental health Trust. The first phase allowed contextual information to be gathered from multiple UK Trusts about how they had implemented the different approaches. This informed phases 2 and 3 and allowed the application of broad findings to a local context. The quantitative online survey was undertaken to gather the wide views of stakeholders while the qualitative focus groups allowed richer, in-depth, contextualised information. Each phase is described in more detail below.

### Setting and access

The study took place between June and October 2019 in Oxford, UK. The Appreciative Inquiry enlisted four additional NHS mental health trusts in South England. We were keen to gain the perspectives of a range of organisations using either the ‘opt-in’ or ‘opt-out’ method and approached seven organisations; four of which use an ‘opt-in’ method and three organisations using an ‘opt-out’ approach. Of these, four chose to participate: one uses the ‘opt-in’ method and the other three organisations have moved to an ‘opt-out’ method.

### Phase 1: Appreciative Inquiry

#### Participants

Participants were recruited using snowball sampling. Initial contact was made with each organisation’s Research and Development (R&D) Department by a member of the research team (J.P.). If willing to participate, the R&D contact was asked to invite other team members, providing they had been involved in implementing ‘opt-in/out’ for their organisation.

#### Procedures

Three of the Appreciative Inquiry discussions took place in rooms at the participating organisations; the fourth took place via telephone. All discussions lasted 60–120 minutes and refreshments were provided at the face-to-face meetings. Each discussion was facilitated by at least two members of the research team (C.H., J.P., S.W.) and used prompts from a semi-structured topic guide. Topic guide questions included the following: ‘Tell us what led to your organisation taking an ‘opt-in/out’ approach?’ and ‘What has been achieved by implementing the ‘opt-in/out’ approach?’ Extensive handwritten notes were taken.

#### Data analysis

Qualitative data were thematically analysed and managed using the Framework approach to enable findings to be collated across and within the Appreciative Inquiry discussions (Gale et al., 2013). Initial codes and categories were grouped and regrouped until themes began to emerge from the data set. Research group meetings (C.H., J.P.) enabled triangulation of the data by ensuring consistency in the interpretation of the data set and collective agreement of the themes generated.

### Phase 2: staff and membership surveys

#### Participants and procedures

Two online surveys were developed at OHFT by the researchers using Survey Monkey. The first explored staff attitudes towards clinical research and the different approaches to informing patients about research. Questions covered topics including how frequently clinicians talked to patients about research opportunities and their confidence in doing so, as well as issues relating to patient capacity. The second survey was developed for the membership of OHFT. This survey explored non-staff members’ perceptions of clinical research. Questions included ‘Do you think patients [at OHFT] expect to be informed about research opportunities?’ and ‘Should all patients be offered the opportunity to participate in research?’ Links to the online survey were made available for staff via the Trust’s intranet and circulated within the Trust’s weekly email update. The membership survey was sent to non-staff members via email, by the Trust communication team.

#### Data analysis

Survey Monkey data from both surveys were exported into an Excel spreadsheet. Descriptive statistics summarised the data; frequencies and percentages were used to report on the surveys’ responses.

### Phases 3: patient and staff focus groups

#### Participants and procedures

##### Patients’ focus groups

Four focus groups were held with OHFT patients from memory clinics, adult mental health teams, acute adult wards, and patient and public involvement (PPI) representatives. Fifty-five patients were contacted by telephone, or in person, by a member of their care team and were invited to participate. Eleven of these patients agreed to participate. The main reason for declining was due to not wanting to take part in a group discussion; however, time and resources meant individual interviews were not feasible. The PPI focus group was arranged by the Trust’s R&D PPI Lead, who emailed 41 PPI members to invite them to attend. Of these, five agreed to participate.

##### Staff focus groups

Five focus groups were held with staff members of OHFT. The teams were contacted by a study researcher (J.P.) and invited to participate. Focus group members provided representation from the following services: community hospitals, a specialist peripatetic community team and two regional adult mental health teams. In addition, the final focus group was made up of Senior Nurses from across the Trust; this was organised through the researchers (J.P., C.H.) contacting the Chief Nurse to ask for their support in enlisting Senior Nurses. All focus groups were held in clinical settings, at times and dates that suited most participants; refreshments were provided. The focus groups were facilitated by two researchers (J.P., S.W. or C.H.), using a semi-structured topic guide, were digitally recorded and lasted for 60 minutes, apart from the focus group on the adult acute ward, which was not recorded because patients did not feel comfortable with this happening; instead, extensive handwritten notes were taken. Patient participants were paid £20 for their involvement and travel expenses were covered.

### Data analysis

The focus group recordings were transcribed by a professional transcription company, with an appropriate confidentiality agreement in place. Data were thematically analysed and managed using the Framework approach. Initial codes and categories were grouped and regrouped until themes began to emerge, and meetings with C.H. and J.P. served as a means of triangulating the data by ensuring consistent interpretation of the data and agreement of the final themes to emerge from the data-set.

### Ethics and consent

The study was a local audit, approved by the Clinical Directors at OHFT; therefore, ethical approvals were not required. However, written informed consent was obtained from all participants prior to participation. The authors assert that all procedures contributing to this work comply with the ethical standards of the relevant national and institutional committees on human experimentation and with the Helsinki Declaration of 1975, as revised in 2008.

## Results

### Phase 1: Appreciative Inquiry

The five participating organisations, including OHFT, varied in size and geographic location, covering both rural and urban populations. All five provided mental health services to children and adults. Three organisations had moved to an ‘opt-out’ method of informing patients about research opportunities, while the other two utilised a clinician-led ‘opt-in’ approach (Table 1).

**Table 1.** Summary of characteristics of participating Appreciative Inquiry organisations.

NHS Trust	Clinical services offered	Employees^a^	Patient population	Approach
Organisation A	Inpatient and Community Mental Health	4000	1.8 million	Opt-out
Organisation B	Inpatient and Community Mental Health; Learning Disability Service	5000	1.8 million	Opt-out
Organisation C	Inpatient and Community Mental Health; Learning Disability Service	2600	890,000	Opt-out
Organisation D	Inpatient and Community Mental Health; Substance misuse service	4600	1.3 million	Opt-in
Organisation E	Inpatient and Community Mental Health; Learning Disability service; Community healthcare	6900	2.5 million	Opt-in

a Rounded to the nearest hundred.

The main themes to emerge from the qualitative data set, and presented in Table 2, were as follows:

- Accessibility of research information;
- Challenges around implementation;
- Data management.

**Table 2.** Summary of themes from Appreciative Inquiry discussions.

Accessibility of research information	Challenges around implementation	Data management
It’s about inclusivity … reaching more people, and people who didn’t realise there were research opportunities (*Organisation B*) It’s a relatively low proportion of the total of our patients, but because the throughput is so much, we’ve managed to build up the register (*Organisation D*)	Information Governance could put a stop to everything, get them involved from the beginning (*Organisation B*) There’s an absolute need for high level support (*Organisation A*) It was important to receive buy-in from the Chief Executive and IG manager early on (*Organisation C*)	Opt-out is a task in the public interest (*Organisation A*) A patient can reasonably expect to hear about research as part of their routine care, and every measure was taken to show that research is part of routine clinical care and patients are sufficiently warned/informed (*Organisation C*) The governance of data is important and something that needs proper consideration. You need to think about what data you can extract, how long you can keep it and where to store it (*Organisation B*)

#### Accessibility of research information

A common aim among participants was for *all* patients to be able to access sufficient information about research opportunities. However, some participants were concerned that the ‘opt-in’ approach was dependent on individual clinicians offering research opportunities to select patients.

#### Challenges around implementation

There were noticeable differences in the practicalities of delivering ‘opt-out’ between organisations. One ‘opt-out’ organisation posted patients ‘opt-out’ information leaflets explaining the ‘opt-out’ approach, before posting out study-specific information. Other organisations sent out study-specific information to patients without prior notification. In addition, high-level support at Executive level was viewed by all organisations as essential for successful implementation, alongside the need for any ‘opt-out’ terminology to be clear and straightforward, to avoid misleading people as to its purpose.

#### Data management

All participants were acutely aware of the need for their organisation’s approach to be fully compliant with the General Data Protection Regulation (GDPR), Europe-wide legislation adopted by the UK Government in 2018 (Data Protection Act, 2018) and the accompanying National Data Opt-Out, a service that allows patients to opt out from their personal data being used for research (National Data Opt-Out, 2020). Opt-out had been confirmed as GDPR compliant by the Information Governance team at each organisation as it is a task carried out in the ‘public interest’, is necessary for ‘scientific or historical research purposes’ and because all patients can ‘reasonably expect to hear about research’.

### Phase 2: surveys

Overall, 333 staff and non-staff members responded to two online surveys.

#### Staff

Among staff, we collected 201 replies: responders had from 2 to over 20 years of clinical experience and were nurses (*n* = 86, 43%), allied health professionals (*n* = 31, 15%), researchers (*n* = 18, 9%), doctors (*n* = 17, 8%), administrative staff (*n* = 5, 2%) and other (*n* = 44, 23%).

A total of 73% of staff (*n* = 147) agreed that they would be supportive of moving to an ‘opt-out’ approach, with only 31% (*n* = 63) routinely speaking to patients about research; 14% (*n* = 29) stated they did not have time to speak with patients about research, while 18% (*n* = 37) stated they did not feel confident discussing research with patients.

#### Non-staff members

Of 980 non-staff members sent the online membership survey, 132 (13%) responded. Overall, 91% (*n* = 120) of non-staff members reported they would be supportive of moving to an ‘opt-out’ approach provided appropriate measures were taken to protect patients, including the provision of clear information about how to opt out, clear communication between researchers and clinical teams and contact with patients only being made after capacity assessments had been undertaken. A total of 81% (*n* = 108) were aware that OHFT was research active and 77% (*n* = 103) thought patients would expect to be informed about research opportunities as part of routine care.

### Phase 3: focus groups

Overall, 19 members of staff and 16 patients and carers participated. A summary of patient and staff characteristics is displayed in Table 3.

**Table 3.** Summary of patient and staff characteristics of focus group participants.

		Memory Clinic participants (n = 4)	Adult Mental Health Team participants (n = 3)	Acute Adult Ward participants (n = 4)	Public and Patient Involvement participants (n = 5)	Staff (n = 19)
Age in years (range)	Patient	68.5 (65–72)	44.3 (29–57)	42.3 (34–47)	56.4 (45–67)	Not known
	Carer	Not known	–	–	–	Not known
Years of clinical experience (range)						21 (2–39)
Gender	Male	2	2	4	2	4
	Female	2	1	0	3	15
Ethnicity	White British	4	2	4	5	17
	Black British	0	1	0	0	1
	White other					1
Clinical diagnoses	Organic, including symptomatic, mental disorders (includes dementia)	2	0	0	Not known	–
	Schizophrenia, schizotypal and delusional disorders	0	2	4	Not known	–
	Disorders of adult personality and behaviour	0	1	0	Not known	–
Participated in research	Yes	2	3	0	2	Not known
	No	0	0	4	3	Not known
Profession	Assistant psychologist					1
	Nurse					11
	Occupational therapist					2
	Social Worker					1
	Physiotherapist					4

The main themes to emerge from the data-set, and presented in Table 4, related to the following:

- Research activity and engagement among staff;
- The exclusivity of research;
- Acceptability and accessibility of the ‘opt-out’ approach.

**Table 4.** Summary of themes from patient and staff focus groups.

Research activity	Acceptability and accessibility of the ‘opt-out’ approach	The exclusivity of research	Research engagement among staff
*My doctor has never asked me to do research which is strange, as you would think it would be the ideal time* (PPI member) ______ *I didn’t realise there was anything happening here* (MC patient) ______ *I wasn’t aware that research happened here* (AMHT patient) ______ *There’s a cultural shift needed … because they [staff] see research as something that doesn’t happen here but happens elsewhere and is quite separate* (SG1)	*I don’t mind a researcher contacting me directly and have a chat on the phone and follow up later* (AMHT) ______ *I want to hear about everything and I make the decision whether it is right for me* (AMHT patient) ______ *I don’t like random [phone] numbers … I would be happy to receive a text, with a number to call if you wanted to ask questions* (AAW patient) ______ *It’s* [opt-out] *an excellent way to do it, because I think not everybody does get asked* (SG1) ______ *My thoughts are how many people are missing out being informed about research by not being asked the question in the first place. That’s my concern* (SG2) ______ *I suppose for me, that’s the key issue, patients need to be very clear they can opt-out* (SG3)	*There is a lack of studies around …. And they [learning disabilities] are a marginalised group* (SG5) ______ *There’s an issue to do with patient inclusivity in research with housebound people for a start … that’s not to say that they can’t be involved in research but the sorts of research methods that are used, are not very inclusive of that group* (SG5)	*I’m not sure it’s high on their* [staff] *list of priorities (SG1)* ______ *Staff do think about it* [research] *but because of competing demands it slips down the list* (SG4) ______ *Right now, there’s such a capacity and demand gap, the nurses don’t have time to even do the visits … research feels like a nice luxury event* (SG5)

AMHT: Adult Mental Health Team; AWW: Acute Adult Ward; MC: Memory Clinic; PPI: Patient and Public Involvement; SG: staff group.

#### Research activity and engagement among staff

Most participants did not view OHFT as research-active, with patients not being offered the opportunity to discuss research with the healthcare professionals they encountered. There was widespread agreement among staff that due to other clinical pressures, research was deprioritised as ‘non-essential’ care.

#### Exclusivity of research

There was a concern raised that certain patient populations were often excluded from research because research methodologies did not lend themselves to the participation of these patients, and because certain patient groups, such as people with learning difficulties, were perceived as being resistant, or difficult to engage, due to difficulties around the informed consent process.

#### Acceptability and accessibility of the ‘opt-out’ approach

Moving to an ‘opt-out’ approach was considered acceptable by all participants. It was felt that ‘opt-out’ would increase research inclusivity, with more patients being offered research opportunities. Some participants raised concerns over the mode of delivery of research opportunities, commenting that this was important in determining their likelihood of learning more about what was available to them for participation. It was considered imperative that patients were clearly informed that they could ‘opt-out’, with clear guidance on how to do so, with one suggestion that this information should be included within routine appointment letters.

## Discussion

Our findings, relating to accessibility of research information and staff engagement with research, suggest that research remains a marginalised part of healthcare and, where patients are reliant upon clinicians to hear about research opportunities, that some patients, especially those deemed ‘hard to reach’, miss out (Patterson et al., 2011). Mental healthcare patients may be particularly vulnerable to being ‘hard to reach’ due to the nature of their diagnosis, trial design and capacity to consent (Patterson et al., 2010). The ‘opt-out’ approach is widely considered acceptable to patients and clinical staff and offers the potential to be more inclusive, allowing all patients the opportunity to hear about relevant research. The implementation of an ‘opt-out’ approach will only be successful, however, if there is support from clinical teams, Information Governance Leads and the Executive Team. Unfortunately, difficulties can be encountered during the set-up and recruitment phases of clinical studies and randomised trials, related to gaining the necessary ethical and governance approvals and applying for NHS costs to undertake and deliver the research (Snooks et al., 2012).

Most participants in our study viewed research as a positive way to enhance clinical care and ‘opt-out’ a way to ensure patients are empowered and given the right level of autonomy to decide as to what extent, if any, they wish to be involved in clinical research. Compared to ‘opt-out’, our findings have illustrated the potential limitations of relying on an ‘opt-in’ approach, which runs the risk of creating an exclusive, rather than inclusive, research agenda, where only a small proportion of patients are offered research opportunities (Borschmann et al., 2014). This exclusivity of access, which is largely dependent on the research interests of individual clinicians caring for patients, goes against the ethical principles of research, which is more concerned with an equity of access (NHS Constitution for England, 2020). Adopting a more inclusive ‘opt-out’ approach may be transformational by allowing all patients the opportunity to hear about relevant research, regardless of their socio-demographic status, psychiatric diagnosis, mental capacity or position in the healthcare system. Furthermore, patients learn more about their treatment and overall health from being part of research (Castillo et al., 2012). By enabling the provision of relevant information about research opportunities to all patients, the likelihood of them making informed decisions about whether to take part is increased as they are fully informed and engaged from the outset. This removes the possibility of some patients feeling coerced into entering a research study based on their clinician’s recommendation, or out of perceived loyalty to them.

The ‘opt-out’ approach reduces the need for research teams to be reliant on research-active clinicians as study gatekeepers. As well as minimising the potential for selective recruitment in this way, due to the paternalistic tendencies of some clinicians, this also has the potential to increase recruitment to studies involving traditionally ‘hard-to-reach’ groups, or those who might otherwise not hear about research due to a lack of awareness or engagement (Rees and Wells, 2010). While the ‘opt-out’ approach removes the gatekeeping role of clinical staff passing on research opportunities to patients, it should be acknowledged that the ‘opt-out’ approach will only be successful with substantial buy-in from clinical care teams. Patients often have long-established, trusting relationships with their care teams and may seek advice and informational support from these teams in relation to any research of which they have been made aware (Patterson et al., 2011). As a result, it is imperative that clinical teams are engaged with the ‘opt-out’ approach from the start and are viewed as key partners in its implementation. This can be achieved through clear communication about the ‘opt-out’ approach at an organisation-wide level, consistent branding and messaging to all patients and staff, regular R&D updates and by research teams continuing to work closely, and build new alliances, with clinical teams across the organisation to enhance relationships and raise the research profile (Cooke et al., 2008).

Our findings have highlighted that there are key challenges involved in implementing an ‘opt-out’ approach, including ensuring the involvement of key stakeholders, such as Chief Executives and Information Governance Leads at an early stage, as well as ensuring that the language used to communicate the opt-out approach to staff is clear, concise and transparent. Close working with the Communications Leads within every organisation can help ensure that this is achieved. In addition, as well as engaging with ‘top-level’ stakeholders, it is also essential that staff across the organisation feel empowered to be involved and included in the transition to an ‘opt-out’ approach (Grol, 2002). This can perhaps be achieved by regular communications updates, R&D events and widespread dissemination of this approach across different services and directorates.

Care and attention must also be given to some of the set-up processes involved in the transition to ‘opt-out’. Issues such as where to record a patient’s decision about opting in or out, and whether patients who have consented to be contacted about research, but who have been recently discharged from the trust, are still able to be contacted, require careful consideration and should involve multiple expert stakeholders (Vermeulen et al., 2009). This can be facilitated through the establishment of a Steering Group committee, which can be used as an expert forum for discussing any issues and challenges as they arise to produce the most suitable solutions.

The legality of the ‘opt-out’ approach in regard to GDPR had been explored thoroughly by the participating organisations, with an agreement that they had moved to the ‘opt-out’ approach because research is a task carried out in public interest, and because of the concept that patients should have a ‘reasonable expectation’ to hear about research opportunities as part of their routine clinical care (Data Protection Act, 2018). However, in implementing an ‘opt-out’ approach care must be taken to ensure that any processes put in place are fully compliant with GDPR and do not breach any of the key mandatory requirements set out by legislation (Data Protection Act, 2018). One method for facilitating this may be through the completion of a Data Protection Impact Assessment, which helps identify and minimise risk where NHS personal data are being used and can establish the likelihood and severity of any impact on individuals (Data Protection Impact Assessment, 2020). Close working with the organisation’s Information Governance Lead, as a key member of the Steering Group Committee, should help guide this process, as well as seeking advice from other organisations who have implemented similar ‘opt-out’ processes.

### Limitations

While every effort was made to include patients’ views in this consultation, it was not possible to speak with patients in community settings, partly because participation in research is not routine within many UK Trusts. This difficulty in engagement is underscored by the discordance between the generally positive view of research and the low response rate in our staff survey, and the fact that we were unable to include a single doctor in any of our focus groups; busy clinical teams lack time for research activity (Rees and Wells, 2010). This study is only representative of mental healthcare settings; however, many of the findings may be transferable to other healthcare settings.

### Implications

Our findings suggest that ‘opt-out’ is more beneficial than an ‘opt-in’ approach for informing patients about research opportunities. Not only does ‘opt-out’ have the potential to vastly increase patient access to research opportunities, it also enables greater equality of information provision for currently marginalised groups, who are not routinely given the opportunity to hear about, or to access, clinical research. This should ensure that healthcare research is more representative of the entire population, including those with a mental health diagnosis, and therefore improve its relevance.

As a result of these findings, the next step is to implement and evaluate the ‘opt-out’ approach at the lead trust, ensuring that relevant data pertaining to recruitment activity and the impact of the ‘opt-out’ on research activity, patient and staff engagement and collaborative working opportunities are collected. Once this has been achieved, it will be important to collate these findings with a view to developing a framework to be shared on a national and international level, to encourage other healthcare organisations to engage with the ‘opt-out’ approach as a means of transforming patient care and the health outcomes of populations. The impetus for this transformation has been highlighted by the recent COVID-19 pandemic, which has demonstrated the need for rapid and widespread recruitment of participants from all sections of society into research across all manner of healthcare settings (Andrews et al., 2018; Fleming et al., 2020).
